# TMT quantitative proteomics reveals key proteins relevant to microRNA-1-mediated regulation in osteoarthritis

**DOI:** 10.1186/s12953-023-00223-8

**Published:** 2023-11-22

**Authors:** Pinpin Jiang, Dan Liang, Hang Wang, Raorao Zhou, Xianda Che, Linlin Cong, Penghua Li, Chunfang Wang, Wenjin Li, Xiaochun Wei, Pengcui Li

**Affiliations:** 1 Department of Orthopaedic Surgery, the Second Hospital of Shanxi Medical University, Taiyuan, 030001 Shanxi China; 2 Key Laboratory of Bone and Soft Tissue Injury, Second Hospital of Shanxi Medical University, Taiyuan, China; 3 Department of Health Statistics, School of Public Health, Shanxi Medical University, Taiyuan, 030001 Shanxi China; 4 College of Basic Medical Sciences, Shanxi Medical University, Taiyuan, China; 5 Department of Laboratory Medicine, Fenyang Hospital Affiliated to Shanxi Medical University, Fenyang, China; 6 Laboratory Animal Center of Shanxi Medical University, Taiyuan, China; 7 Department of Stomatology, the Second Hospital of Shanxi Medical University, Taiyuan, 030001 Shanxi China

**Keywords:** Osteoarthritis, Proteomics, Differentially expressed proteins, Fn1, P4ha1, Acan

## Abstract

Osteoarthritis (OA) is the second-commonest arthritis, but pathogenic and regulatory mechanisms underlying OA remain incompletely understood. Here, we aimed to identify the mechanisms associated with microRNA-1 (miR-1) treatment of OA in rodent OA models using a proteomic approach. First, *N* = 18 Sprague Dawley (SD) rats underwent sham surgery (*n* = 6) or ACL transection (*n* = 12), followed at an interval of one week by randomization of the ACL transection group to intra-articular administration of either 50 µL placebo (control group) or miR-1 agomir, a mimic of endogenous miR-1 (experimental group). After allowing for eight weeks of remodeling, articular cartilage tissue was harvested and immunohistochemically stained for the presence of MMP-13. Second, *N* = 30 Col2a1-cre-ERT2 /GFPf1/fl -RFP-miR-1 transgenic mice were randomized to intra-articular administration of either placebo (control group, *N* = 15) or tamoxifen, an inducer of miR-1 expression (experimental group, *N* = 15), before undergoing surgical disruption of the medial meniscus (DMM) after an interval of five days. After allowing for eight weeks of remodeling, articular cartilage tissue was harvested and underwent differential proteomic analysis. Specifically, tandem mass tagging (TMT) quantitative proteomic analysis was employed to identify inter-group differentially-expressed proteins (DEP), and selected DEPs were validated using real-time quantitative polymerase chain reaction (RT-qPCR) technology. Immunohistochemically-detected MMP-13 expression was significantly lower in the experimental rat group, and proteomic analyses of mouse tissue homogenate demonstrated that of 3526 identified proteins, 345 were differentially expressed (relative up- and down-regulation) in the experimental group. Proteins Fn1, P4ha1, P4ha2, Acan, F2, Col3a1, Fga, Rps29, Rpl34, and Fgg were the *top ten most-connected proteins, implying that miR-1 may regulate an expression network involving these proteins. Of these ten proteins, three were selected for further validation by RT-qPCR: the transcript of Fn1, known to be associated with OA, exhibited relative upregulation in the experimental group, whereas the transcripts of P4ha1 and Acan exhibited relative downregulation. These proteins may thus represent key miR-1 targets during OA-regulatory mechanisms, and may provide additional insights regarding therapeutic mechanisms of miR-1 in context of OA.

## Introduction

Osteoarthritis (OA) can causes stiffness, arthralgia, and even disability. The incidence of OA gradually increases with age, OA is the commonest type of arthritis. According to epidemiological statistics, more than 32.5 million adults in the United States have OA, which has a significant impact on the health of society and patient quality of life [1]. Osteoarthritis is characterized by thinning of cartilage thickness, osteophytes formation, and chondrocyte reduction [2]. Due to the lack of blood supply and nerve control in the articular cartilage, it is often not effectively repaired after injury. Although there is as yet no convincing evidence of OA modification or reversal, but several ongoing clinical trials are in the process of assessing potential novel disease-modifying interventions [3]. RNA-based therapeutic interventions are an established area of research in OA.

In humans, OA occurs in up to 87% of patients following reconstructive surgery for anterior cruciate ligament (ACL) trauma [4]. While ACL transection is often used in rats to produce a preclinical OA model, in mice the less invasive surgical destabilization of the medial meniscus (DMM) is preferred and induces detectable OA as early as two weeks following surgery [5].

MicroRNAs (miRNAs) are small non-coding RNA molecules of about 20–25 bp in size that are transcribed by RNA polymerase II and expressed endogenously in eukaryotes [6]. Micro RNAs can bind to the 3’-UTR of mRNA through base-pairing to regulate the post-transcriptional translation of genes. Previous studies have suggested that miRNAs can participate in slowing of OA progression by regulating chondrocyte proliferation, apoptosis, and extracellular matrix (ECM) metabolism [7]. miR-214–3p inhibited the ECM catabolism and chondrocyte apoptosis by targeting IKKβ; miR-27b-3p has a key role in ECM regulation associated with synovial fibrosis during OA [8, 9]. The miRNA agomir is a special chemically modified miRNA agonist that mimics endogenous miRNA to inhibit the expression of target gene mRNA. H.-b. Si et al. found that intra-articular injection of miR140 agomir delayed the progression of osteoarthritis by modulating ECM homeostasis in rat chondrocytes [10]. It has been shown that miR-1 can reduce cartilage damage caused by OA by regulating the IHH pathway [11], and miR-1 can regulate the chondrocyte phenotype through the Wnt/β-catenin pathway [12].

Mass spectrometry-based proteomics in concert with advanced bioinformatic techniques to study dynamic changes in protein composition and expression levels using a high-resolution, high-precision approach [13]. Such proteomic techniques can identify disease-associated proteomic signatures, as well as facilitating deeper exploration of pathogenic mechanisms.

Therefore, we investigated whether miR-1 small nucleic acid drugs could slow down the progression of OA by intra-articular injection in rats. Subsequently, by establishing mice with the Col2a1-cre-ER^T2^ /GFP^f1/fl^ -RFP-miR-1 gene, proteomics and bioinformatics analysis of mouse cartilage using tandem mass tagging (TMT) labeling combined with high-resolution LC-MS/MS was performed to explore the differential proteins after induction of OA mice with overexpressing the miR-1 gene, followed by validation of the expression levels of key genes using real-time quantitative polymerase chain reaction (RT–qPCR). Findings of this study contribute to validating the potential therapeutic role of miR-1 in OA, as well as helping to elucidate molecular mechanisms underlying this action. Results are particularly significant in context of the urgent need for disease-preventive or -modifying interventions in OA.

## Materials and methods

### Animals and care

The experimental animals used in this study were approved by the Animal Protection and Utilization Committee of the Second Hospital of Shanxi Medical University (Taiyuan, China) (2021010,DW2022063)and strictly adhered to the Guidelines for the Protection and Use of Laboratory Animals issued by the China Animal Science Council. A total of 18 Sprague Dawley (SD) rats and 30 mices with the Col2a1-cre-ER^T2^ /GFP^f1/fl^ -RFP-miR-1 gene were housed in specific pathogen-free (SPF) barrier facilities under controlled temperature and humidity with alternating 12-h light and dark cycles. Experimental animals were fed SPF-grade chow and sterile drinking water. The behavior and health status of rats and mice were monitored regularly during the experiment. Animals were anesthetized with sodium pentobarbital.

### miR-1 small nucleic acid drugs

miR-1 small nucleic acid drugs (miRNA agomir) were supplied by the Guangzhou RIBOBIO Corporation (Guangzhou, China). miRNA agonists regulate target mRNA levels by mimicking endogenous miRNAs.

### Animals modeling

Eighteen 8-week-old SD rats were equally divided into three groups: sham-operated, negative control, and experimental groups. Only the joint capsule was cut in the sham-operated group, while the negative control group and experimental group underwent anterior cruciate ligament transection (ACLT). One week after surgery, 50 µL saline was injected into the joint cavity of the sham-operated group, 50 µL agomiR-1 control was injected into the negative control group, and 50 µL agomiR-1 small nucleic acid drug was injected into the experimental group [14, 15]. The rats were euthanized and the articular cartilage tissue was removed from each group of SD rats 8 weeks after modelling. The Col2a1-cre-ERT2 /GFPf1/fl -RFP-miR-1 gene mice were randomly divided into experimental and control groups and the experimental mice were injected with tamoxifen (Sigma Aldrich, USA), 100 uL, at a concentration of 20 mg/mL for 5 d at 8 weeks, while the control mice were injected with only an equal amount of corn oil. Fifteen days later, the experimental and control groups were simultaneously underwent destabilization of medial meniscus (DMM) to induce OA in the knee of mice [16]. After 8 weeks of modeling, the mice were euthanized and the articular cartilage was removed.

### Immunohistochemistry

Paraffin specimens were serially sectioned 5 μm thick, followed by immunohistochemical staining to detect the expression levels of target proteins, followed by the primary antibody: rabbit anti-mouse anti-MMP-13 (1:100 cat. no. bs-10581R, Bioss).

### TMT-based proteomics of knee cartilage

#### Extraction and digestion of cartilage proteins from knee joints

Mouse knee cartilage tissue was well ground in liquid nitrogen, followed by the addition of phenol extraction solution and centrifugation. The upper layer was added to 5 times the volume of pre-cooled 0.1 M ammonium acetate-methanol solution. After centrifugation, the precipitate was washed with methanol, followed by removal of the methanol with acetone and centrifugation. The sample lysate was added to dissolve the precipitate and the protein concentration was determined using a BCA protein assay kit (PC0020, Solarbio, Beijing, China). DTT at a concentration of 5 mM was added to the sample, incubated for 30 min and the same volume of iodoacetamide was added, followed by acetone at a ratio of 1:6. The sample was centrifuged and the precipitate was added to 100 µL TEAB (200 mM) and digested overnight with 1/50 of the sample mass of trypsin.

#### TMT labeling and high-performance liquid chromatography (HPLC) fractionation

After tryptic digestion, peptides were reconstituted in TEAB (pH6.5)and labeled using a TMT kit (90066B, ThermoFisher, Shanghai, China). The labeled peptides were fractionated using high-performance reverse liquid chromatography (HPLC) system (Thermo Fisher Scientific, Waltham, MA, USA). The peptides were graded on an Agilent Zorbax Extend - C18 narrow diameter column (Agilent Technologies Co. Ltd, Beijing, China) and dried under vacuum.

#### LC-MS/MS analysis

The peptides were sampled at a flow rate of 300 nL/min onto a pre-column Acclaim PepMap100 100 μm × 2 cm (RP-C18, Thermo Fisher Scientific) and then separated using an analytical column (Acclaim PepMap RSLC, 75 μm × 15 cm; RP-C18, Thermo Fisher). The peptides were analysed by Thermo Scientific Q Exactive mass spectrometer (Thermo Fisher Scientific) and the intact peptides were detected using Orbitrap at 70000 resolution.

MS/MS spectrum collection is completed using a data-dependent positive ion mode for high-energy collision fragmentation with MS/MS resolution set to 17500.

#### Raw data analysis

Proteome Discoverer 2.4 (Thermo Fisher Scientific) was used to identify and analyze the MS/MS raw data. Carbamidomethyl (C), TMT6plex (N⁃ term) were selected as fixed modifications and oxidation (M) as variable modifications; up to 2 missed cuts were allowed for the digested peptide fragments, and the error range was controlled within 10 ppm for the parent ion and 0.02 Da for the peptide ion fragments. The error range is within 10 ppm for the parent ion and 0.02 Da for the peptide ion fragment.

#### Bioinformatics analysis

Candidate differentially-expressed proteins (DEPs) were identified using 1.2-fold change and *p* < 0.05 thresholds, followed by analysis of DEPs using bioinformatics tools. Heat maps and volcano plots of differentially expressed proteins were plotted using R software [17]. Principal component analysis (PCA) of differential proteins was subsequently performed using SPSS (v.26.0) software. Gene Ontology (GO) and Kyoto Encyclopedia of Genes and Genomes (KEGG) pathway enrichment analysis of differentially expressed proteins was conducted using the “clusterProfiler” and “DOSE” tools of R software [18–20]. Protein-protein interaction network analysis was performed using STRING online, and visualization of the network was performed using Cytoscape (v.3.6.0) software [21, 22].

### Primary chondrocyte isolation, culture, and RT-qPCR

Remove the mouse knee cartilage tissue with a blade, frozen it in liquid nitrogen, and ground, and RNA was extracted using the RNAeasy™ Animal RNA Isolation Kit with Spin Column (R0026,beyotime,shanghai,china) according to the manufacturer’s instructions. A portion of the samples were reverse transcribed into cDNA using the HyperScript™III miRNA 1st strand cDNA Synthesis Kit (by stem-loop) (R601, EnzyArtisan, Shanghai, China). The RNA was subsequently reverse transcribed into cDNA and the expression of the target gene in the cDNA was detected by RT-qPCR as previously described [23]. The qPCR conditions were as follows: preincubation of samples at 95 °C for 30 s, and then 40 cycles of denaturation at 95 °C for 15 s, annealing at 60 °C for 45 s, and dissolving at 95 °C for 15 s, 60 °C for 1 min, and 95 °C for 15 s. miRNA-1 qPCR conditions were as follows: Preincubation of samples at 95 °C for 30 s, and then 40 cycles of denaturation at 95 °C for 10 s, annealing at 60 °C for 30 s, and dissolved at 95 °C for 15 s, 60 °C for 1 min, and 95 °C for 15 s.Primer sequences for RT-qPCR were synthesized by Shanghai Biotech Shanghai China, and the sequences are shown in Table 1.

**Table 1:** Primers for used for RT-qPCR

Species	Gene	DNA sequence
Mouse	miR-1-3p	5′-GCGCGTGGAATGTAAAGAAGT-3′
		5′ -AGTGCAGGGTCCGAGGTATT-3′
	Fn1	5′-ACCCGTTTTCATCCAACAAGAG-3′
		5′-CGGTATCCAGACACCACACTATCA-3′
	P4ha1	5’-ATGACCCTGAGACTGGAAA-3’
		5’-GCCAGGCACTCTTAGATACT-3’
	Acan	5’CAGTGGGATGCAGGCTGGCT-3’
		5’CCTCCGGCACTCGTTGGCTG-3’
	18s	5′-CGGCTACCACATCCAAGGAA-3′
		5′-GCTGGAATTACCGCGGCT-3′
	U6	5′-GCTCGCTTCGGCAGCACATATAC-3′
		5′-AGTGCAGGGTCCGAGGTATT-3′

### Statistics

The data is expressed as mean ± standard deviation and statistically analyzed using SPSS software (version 22.0) and Graph Prism software (version 8.0). Results were compared with an independent t-test., where * represents *P* < 0.05 and is statistically significant.

## Results

### Immunohistochemical results

**Fig. 1:**
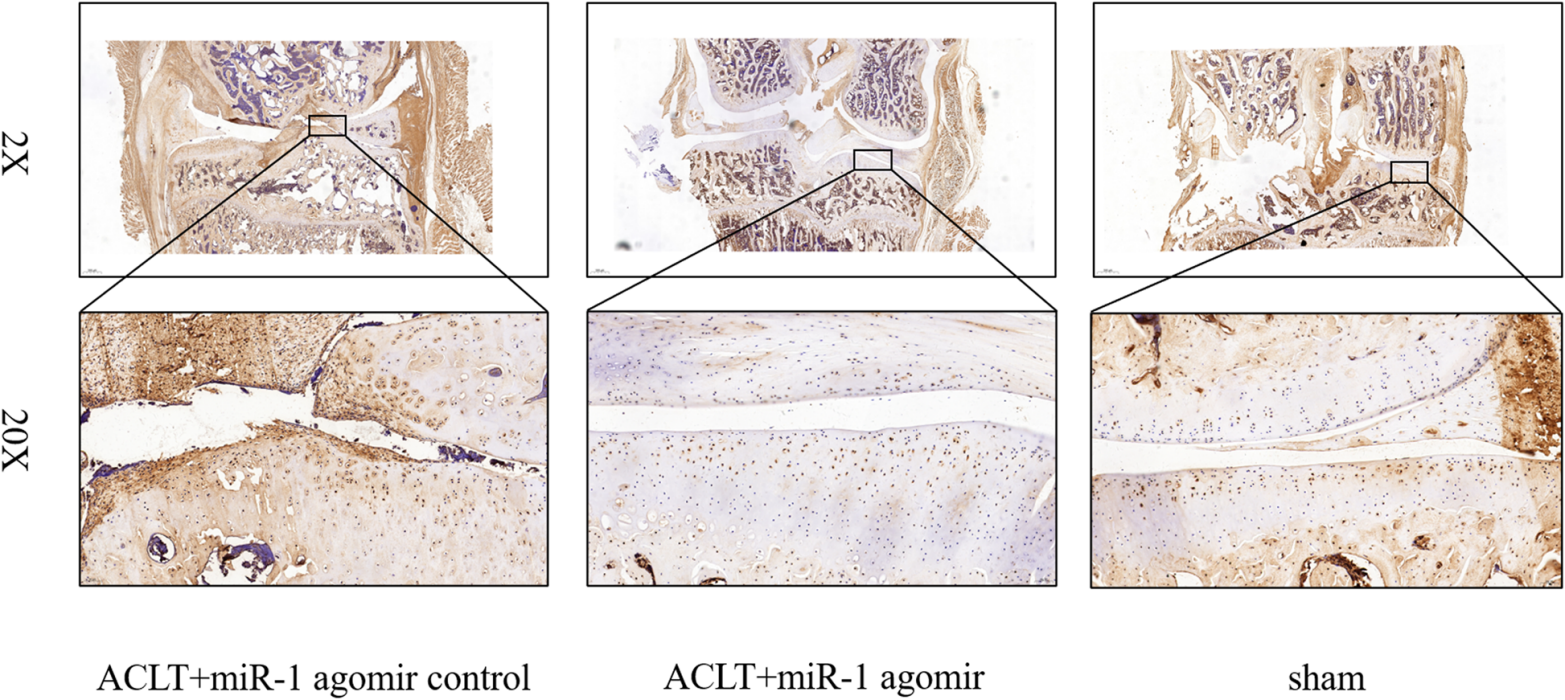
MMP-13 protein expression in rat articular cartilage. (Scale bars are 100 μm and 20 μm)

In a rat OA model, joint cavity injection of miR-1 small nucleic acid drugs inhibited the inflammatory response in OA. Immunohistochemical results showed that MMP-13 protein expression levels in the miR-1 small nucleic acid drug injection group was significantly lower than that in the negative control group (Fig. 1).

### Differentially expressed protein quantification and screening

**Fig. 2:**
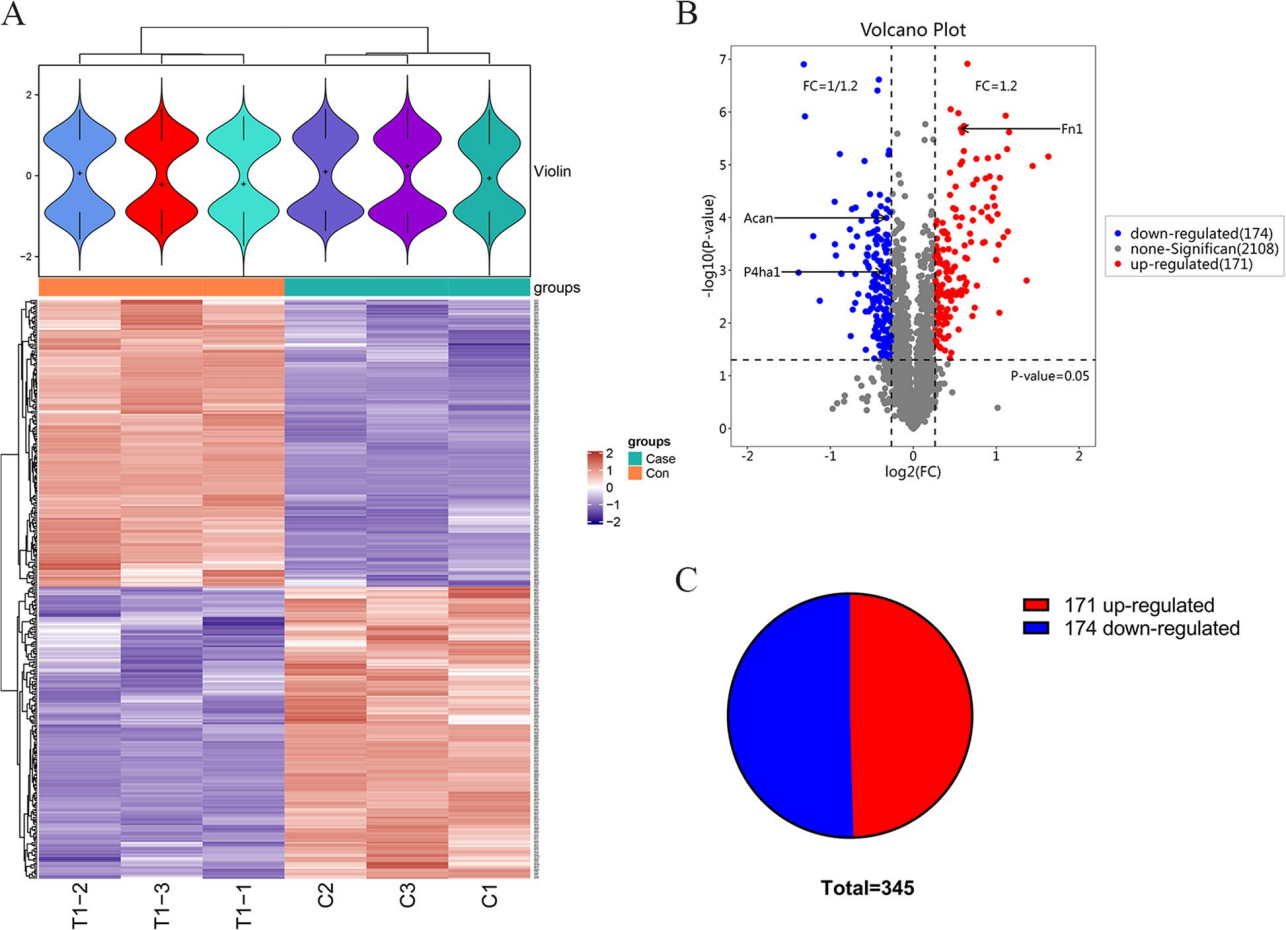
Cluster analysis and volcano plot of candidate DEPs. **A** Each horizontal group represents one protein, and each vertical group represents an independent sample. The proteins are divided into two categories and the samples are divided into two groups based on mathematical expression values. **B** Volcano plots are plotted using the difference ploidy (Log_2_) and P-value (–Log_10_) of the DEPs, with red representing up-regulated proteins, blue representing down-regulated proteins, and gray representing proteins with no significant expression differences. **C** Pie chart showing the proportion of up- and down-regulated differential proteins

A total of 3526 proteins were identified, of which 345 were differentially-expressed. Specific differential protein information is shown in Table 2. A total of 345 DEPs were clustered and analyzed (Fig. 2A). The distribution of all differentially expressed proteins was depicted by the p-value and abundance value ratio in the volcano plot (Fig. 2B). A total of 345 candidate DEPs were identified, of which 171 were upregulated and 174 were downregulated DEPs (Fig. 2C).

**Table 2:** Differentially expressed genes

Differential Expression	Proteins
Downregulated	Col12a1, Col2a1, Epx, Myh10, Serpinh1, Matn1, Acan, Hnrnpm, Cspg4, Col14a1, Serpinf1, Krt10, Col9a1, Hsp90b1, Bgn, Mki67, Atpla2, Papss2, Matn4, Rnf213, Postn, Alox15, Fbn2, Tnn, Top2b, Tmpo, Fkbp9, Ugdh, Prdx5, Pfn1, Snx2, Rbmx, Kera, Krt2, Cmpk2, P4ha2, Smarcc1, Ear1, Prg2, Myoz1, Eif3e, Ces1c. Man2b1, Mthfd1l, Nt5c, Gaa, Igf2r, Ear2, Mmp9, Krt17, Krt6a, Cpox, Niban2, Pdlim7, Pafah1b3, Edil3, Myot, Parp1, Ncl, Eif3d, Sting1, QPdcd4, P4ha1 Nxf1, Mbl2, Lig1, Ptprc, Vamp3, Mepe, App, Sri, Col6a5, P3h3, Dync1li1, Matn2, Mpi, Ahsp, My14, Spp1, Crtap, Srsf3, S1c27a4, Parva, Pdlim5, Nmral1, Ogt. Raly, Ppa1, Dcun1d1, Hnrnpc), Rnm12, IKzf1, Nnt, Ube216, Tap1, Limd2, Snu13, Pym1, Ssr1, Col3a1, Gfpt2, Scara3, Kif4, Adar, Fam50a, Agk, Is1r, Co1ga1t2, Xrn1, Stk38, Ctdsp1, Lsm2, Trim21, Ubq1n2, Usp39, Cstf2, Irgm1, Fam107b, C1qc, S100a6, Exosc10, Samd91, C1qa, Sp110, Tsn, Hdac6, Rangap1, Efemp2 Ifit1, Dmp1, Lsm14a, Mtfr1l, Wbp11, Cpm, Tomm20, Safb2, Ndrg2, Lect2, Traf6, Tardbp, Palm, Col4a3, Grk6, Nexn, Ergic1, Akap12, Mapre3, Golm2, Farp1 Asrgl1, S100a11, Gmeb1, Gzma, Anp32b, Dctn3, Poldip3, Ccdc8, Ddt, Tcn2, il17b, Poglut3, Gng5, Hrnr, Ccdc9, Pold3, Cox6c, Stxbp6, Mrpl45, Camsap1 Pyhinl, Cggbp1, Brk1, Vapb, Rnps1
Upregulated	Fnl, Comp, Apoe, Col6a1, Cilp2, Krt34, Hapln1, Col6a2, Cilp, Ftl1, Pnp, Ogn, Anxa3, Krt81, Fgb, Prelp, Fga, Sparc, Pgml, Thbs4, Ctsg, Krt87, Myl9, Itih4 Angptl7, Serpinb6, Cp, Arpc2, Clu, Gstml, Ighm, Itga2b, Ahsg, Tgm2, F2, Hba, F7, Vtn, Serpina3m, Mup17, 0lfm4, Mmp-13, Mgp, F10, Itih1, Cndp2, Prg4 Capza1, Ehd3, Chad, Prosl, Fgg, Ighg3, Fth1, Igh-3, Apoa4, Gstm2, Clec11a, F13a1, Lgmn, Pzp, Vdac1, Pdhb, Ak2, Dpt, Arhgdia, Atp6v1c1, Rpl18a, Lyz1 Serpina1d, Myoc, Pafah1b1, Vat1, Cyp1b1, Chil4, Npl, Lum, Spp2, Ca3, Lama2, Uchl3, Kv3a8, Htral, Cal, Usp4, Nt5e, Mug1, Gnpdal, Arl8a, Sts, Aspn, Cbr3 Igfbp5, Clptml, Rps29, Cryab, Lsm3, Pvalb, Saal, Binl, Zyx, Retnlg, Eif5b, Atp6v0a1, Mqo2, Apcs, Saa2, Timm50, A2m, Gpx3, Hp, Rplp2, Ctsc, Cd51, Nme1 Crispld2, Kv2a7, Itih3, Abcd2, Fabp5, Ap3d1, Tgfb1, Vps45, C8a, Acad8, Sptlc2, Acox3, BDH1, Sod3, Eef1a2, Arrb1, Pon3, Polr2b, Tkfc, Nptn, Idh2 Aldh3a1, Sect. 63, Papolg, Pld3, Rpl36, Rps27l, Rpl34, Cnbp, Pcmt1, Ubl5, Adh1, Cdh11, Pdk3, Saa4, Igk-V19-17, Tbca, Proc, Mup20, Scpep1, Apom, Galnt1 Slpi, Kv5a5, Gca, Rpl35, Aldh1a2, Arrdc2, Sec11c, Dnajb6, Nsd2, Il11, Mrps27, Bglap2, Bicd1, Reg3b

### PCA

**Fig. 3:**
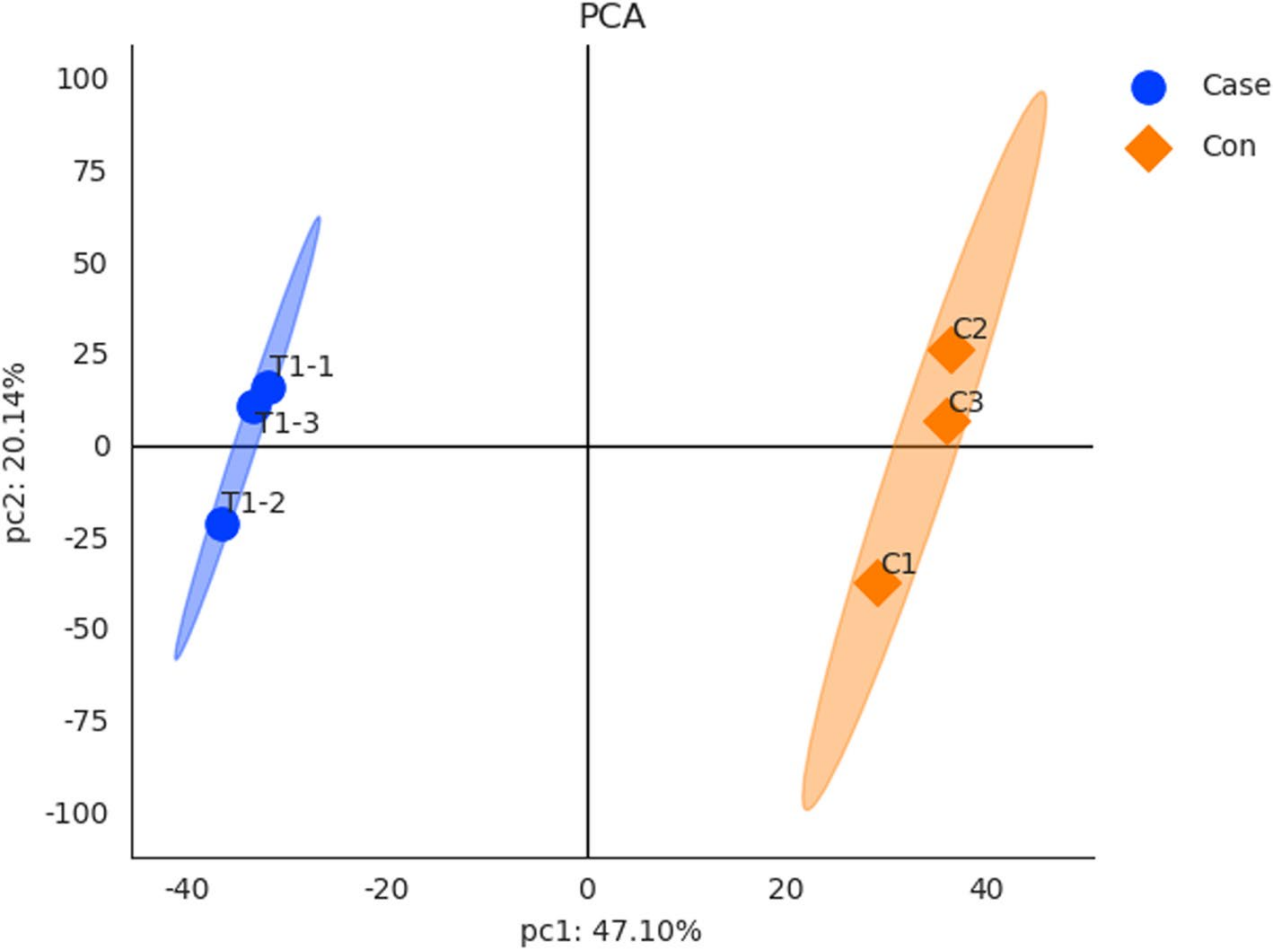
PCA biplot

This study analyzed protein expression in articular cartilage tissue of miR-1-overexpressing mice versus control mice, and defined all proteins as variables in PCA. The PCA score is shown in Fig. 3. PC1 and PC2 explain 47.10% and 20.14% of data variability, respectively. Clear separate clustering of the experimental and control group samples on the basis of proteomic data indicate likely differential expression between these groups.

### GO function and KEGG pathway enrichment analysis of differential proteins

**Fig. 4:**
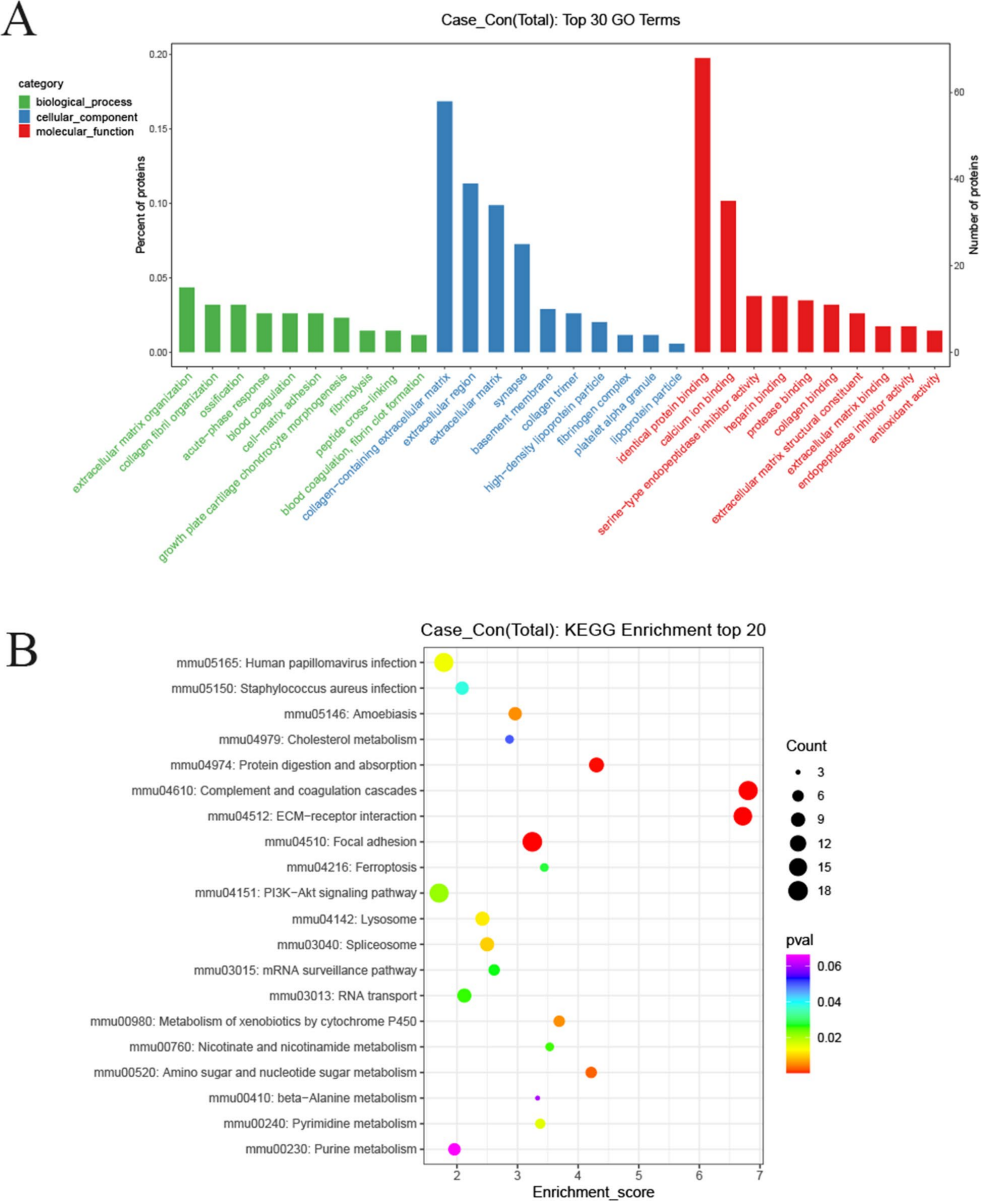
GO (**A**) and KEGG (**B**) analysis of DEPs. The 345 proteins with differential expression were analyzed according to biological processes, cellular components and molecular functions, followed by KEGG analysis. Vertical coordinates in **A** represent the percentage of DEPs in each functional classification. Vertical coordinates in **B** represent the pathways significantly enriched in differential proteins, and horizontal coordinates represent the enrichment score. Larger bubbles indicate more enriched proteins, and redder colors indicate smaller *p*-values

To further analyze the function of differential proteins, we enriched DEPs in terms of biological processes, cellular components, and molecular functions and listed the top 10 entries in each section (as shown in Fig. 4A). GO enrichment analysis of the DEPs showed that biological processes mainly involved ECM organization, collagen fibril organization, and ossification. Cellular components mainly involved collagen-containing ECM, extracellular zone, and ECM, and mainly involved the same protein binding, calcium binding, and serine-type endopeptidase inhibitor activity. The disruption of ECM homeostasis is closely related to OA. Study has shown that miRNA-140 can alleviate OA progression by regulating ECM homeostasis in rats [10]. KEGG pathway analysis was then performed on the DEPs, and the first 20 pathways of the DEPs are shown in Fig. 4. DEPs were mainly enriched in complement and coagulation cascade pathways, ECM-receptor interactions, and adherent spots, as shown in Fig. 4B.

### PPI analysis of the differentially expressed proteins

**Fig. 5:**
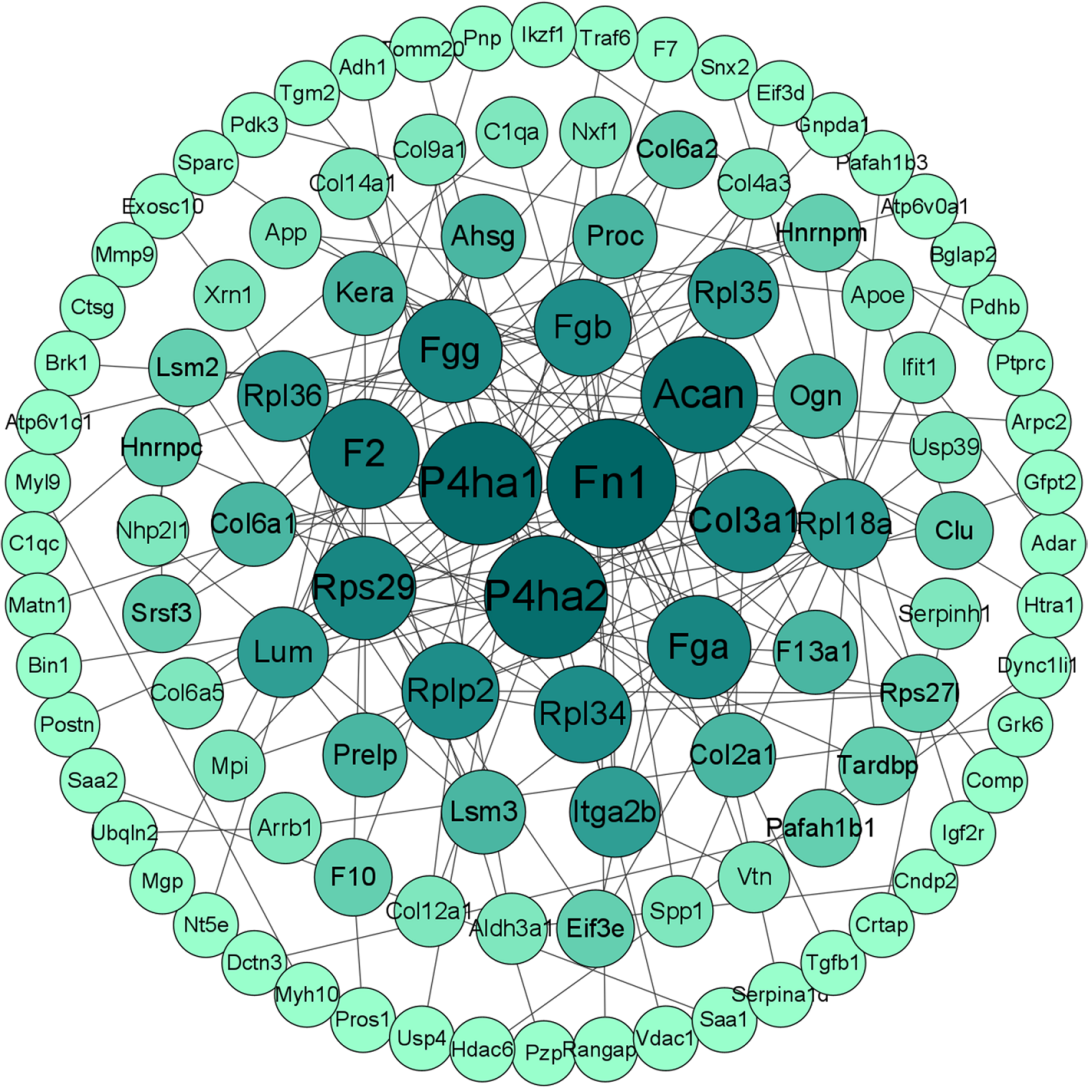
Protein-protein interaction (PPI) of differential proteins. Proteins are indicated by different colored circles, with larger circles and darker colors indicating a higher degree of protein interaction. Protein interactions are illustrated by black lines

Three hundred and forty-five DEPs were analyzed using the STRING online platform to obtain the interaction network maps of the differential proteins. The network graphs were imported into the Cytoscape software for network graph analysis and image processing. The interaction score was used with a threshold of 0.9 (Fig. 5). The analysis revealed that the top ten proteins in terms of network node degree were Fn1, P4ha1, P4ha2, Acan, F2, Col3a1, Fga, Rps29, and Fgg. Previous studies found that Fn1, P4ha1, and Acan were associated with OA. We selected three proteins, Fn1, P4ha1, and Acan, for subsequent validation [24, 25].

### RT-qPCR validation based on TMT results

**Fig. 6:**
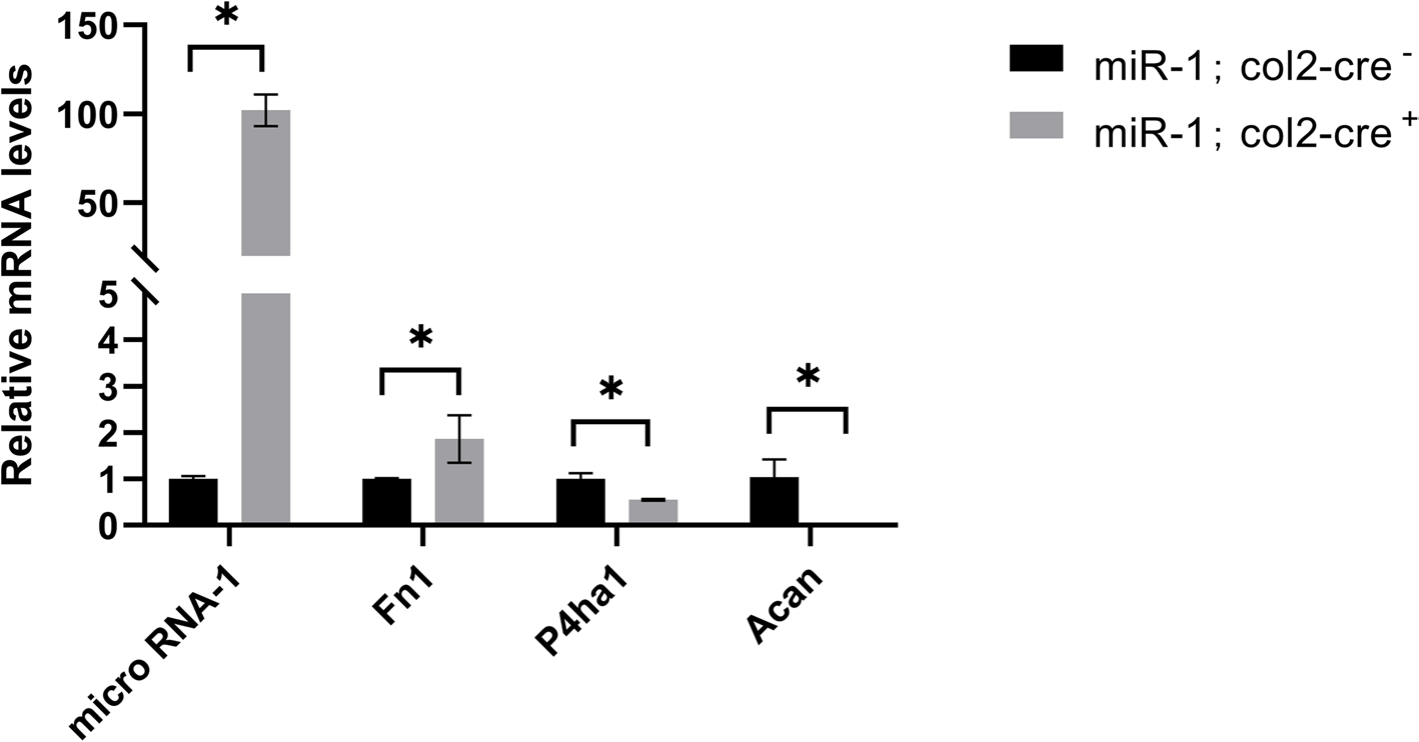
RT-qPCR analysis of the following genes. The mRNA expression levels of Fn1, P4HA1, and Acan after upregulation of miR-1 were consistent with the trend in bioinformatics analysis. *p < 0.05

The RT-qPCR results showed that the expression of miR-1 in the experimental group was significantly higher than that in the control group. The expression of Acan and P4ha1 in the experimental group was lower than that in the control group, while the expression of Fn1 was higher than that in the control group (Fig. 6).

## Discussion

Existing studies have shown that when OA occurs, there are changes in the expression levels of various miRNAs, including miRNA-1 and miRNA-214 [9, 12]. It has been shown that miRNA-1 plays an important role in regulating chondrocyte hypertrophy by inhibiting cartilage catabolism and promoting cartilage matrix synthesis through the Indian hedgehog signaling pathway;in addition, miRNA-1 can also inhibit the Wnt/β-catenin pathway and cartilage degradation metabolism by regulating the expression of FZD7 [11, 12]. However, the pathogenesis of OA has not been fully elucidated; therefore, whether miRNA-1 is involved in the regulation of OA through other pathways deserves further investigation.

In the present study, immunohistochemical results showed that SD rats injected with miRNA-1 small nucleic acid drugs reduced the expression of the inflammatory response marker MMP-13. We then analyzed the cartilage tissues of Col2a1-cre-ER^T2^ /GFP^f1/fl^ /GFP-miR-1 gene mice during OA using a TMT-based quantitative proteomics technique to further explore the regulatory pathways of miRNA-1 in OA. Proteomic analysis revealed 171 proteins with increased expression and 174 proteins with decreased expression in the cartilage tissues of miRNA-1 overexpressing transgenic mice compared with that in control mice. The PPI network was constructed by KEGG enrichment analysis of the differentially expressed proteins, based on STRING online and using the Cytoscape software. Finally, we selected three differentially expressed proteins, Fn1, P4ha2, and Acan, for validation. In addition, upregulating genes such as Prg4 and A2m can effectively inhibit the progression of osteoarthritis. Downregulated genes such as Postn and Mmp9 are negative indicators, and their increased expression promotes the progression of osteoarthritis. Many of the upregulated and downregulated genes have not been confirmed to be related to osteoarthritis, indicating that they may be new intervention targets or research points for osteoarthritis.

Fibronectin 1 (Fn1) is a functional glycoprotein expressed in the chondrocyte membrane and is involved in cell-matrix adhesion. Cartilage tissue is composed of chondrocytes and ECM, and apoptosis of chondrocytes and degradation of the ECM play an important role in the development and progression of OA [26, 27]. Previous studies have shown that Fn1 plays an important role in cartilage regeneration by activating chondrogenic progenitor cells (CPCs) through an intrinsic α5β1-dependent pathway. In addition, Fn1 promotes the accumulation of type II collagen (Col2A1) and proteoglycans on the cartilage surface via the TGF-β/PI3K/Akt pathway [28]. Additionally, Fn1 can inhibit chondrocyte apoptosis by decreasing the expression of p-PI3K/PI3K and p-AKT [29]. The present study confirmed that the expression of Fn1 was significantly higher in the experimental group than that in the control group, which may be an important target for the ability of miRNA-1 to delay OA progression.

Owing to the lack of blood supply to the articular cartilage and the relative confinement of the joint cavity, the articular cartilage tissue is in a hypoxic environment. Proline 4-hydroxylase subunit α1 (P4ha1) is the rate-limiting subunit of proline 4-hydroxylase and is required for the synthesis of various collagen proteins [30]. It has been reported that hypoxia induces P4ha1 expression, which regulates the deposition of collagen in the ECM. In addition, the development of OA is accompanied by the invasion of subchondral bone and deep blood vessels, leading to the development of pain and bone flab formation, P4ha1 can also inhibit neovascularization by inhibiting collagen synthesis, thus disrupting the structure of the vascular basement membrane [31]. Studies have confirmed that the expression of P4ha1 is upregulated in the development of OA [32]. The ECM is not only the living environment for chondrocytes but also the place where chondrocytes exchange signals with the outside world [9]. It is also the site of signal exchange between chondrocytes and the external environment. Proteoglycans (aggrecan), encoded by ACAN, are the main components of the ECM and load-bearing joint. In OA, proteoglycans are degraded by matrix metalloproteinases and proteoglycanases [33]. In our study, we found that P4ha1 and ACAN expression levels were decreased in the experimental group, probably due to the existence of an attempted regeneration process in the early OA [34]. When miRNA-1 was used for treatment, it delayed articular cartilage degeneration, which attenuated gene synthesis and resulted in lower levels of P4ha1 and ACAN expression.

Our study has some limitations, including the small sample size and lack of large-scale experimental validation, as well as the specific mechanism by which miRNA-1 affects the expression of Fn1, P4ha1, and Acan, which requires further in-depth studies in the future.

In summary, we verified that miRNA-1 could delay the progression of OA, and the molecular mechanism of miRNA-1 regulation in OA could be further explored by proteomic analysis. Based on the proteomics and RT-qPCR results, we speculate that miRNA-1 may regulate OA by affecting the expression of Fn1, P4ha1, and Acan.

## Data Availability

All data generated or analyzed during this study are included in this published article.
